# Recent Advances in the Epidemiology of Pathogenic Agents

**DOI:** 10.3390/pathogens13030263

**Published:** 2024-03-19

**Authors:** Wei-Chuan Chen, Yusen Eason Lin

**Affiliations:** 1 Department of Medical Education and Research, Kaohsiung Veterans General Hospital, Kaohsiung 813414, Taiwan; wcchen1027@vghks.gov.tw; 2 School of Medicine, College of Medicine, I-Shou University, Kaohsiung 824, Taiwan; 3 Department of Pharmacy and Master Program, Tajen University, Yanpu Township, Pingtung County 90741, Taiwan; 4 Graduate Institute of Human Resource and Knowledge Management, National Kaohsiung Normal University, Kaohsiung 802561, Taiwan

The COVID-19 pandemic has underscored the pivotal role of epidemiology in studying pathogenic agents. As a scientific discipline that investigates the patterns, causes, and effects of health and disease conditions in specific populations, epidemiology has been instrumental in shaping the global response to the pandemic [1]. The emergence of SARS-CoV-2, the virus responsible for COVID-19, has highlighted the urgent need for robust epidemiological surveillance and research to understand disease transmission dynamics, inform public health interventions, and protect global health [2]. The pandemic has served as a stark reminder of the power of epidemiology as a scientific discipline and a vital tool in our collective arsenal against infectious diseases [1]. As we navigate the post-COVID-19 world, studying the epidemiology of pathogenic agents will remain paramount [1,2].

This Special Issue of the “Epidemiology of Pathogenic Agents” highlighted important articles in recent advances, as shown below.

## 1. Antibiotic Resistance and Emerging Diseases

These studies on the epidemiology of pathogenic agents have shed light on the critical issues of antibiotic resistance and emerging diseases. A global systematic review and meta-analysis revealed a resurgence of syphilis, a sexually transmitted disease that was once on the brink of elimination [Contribution 1]. The study highlighted the global spread of antibiotic resistance, posing significant challenges for the treatment of syphilis. This underscores the need for improved diagnostic methods, treatment strategies, and ongoing research into the development of a vaccine for syphilis. In Taiwan, a study reported a high prevalence of rapidly growing mycobacteria (RGM) infections, with high resistance rates to available antimicrobial agents [Contribution 2]. The findings highlight the need for timely antimicrobial susceptibility testing to guide the selection of appropriate treatment for RGM infections. A study conducted in Mexico found a high prevalence of Helicobacter pylori among patients with gastric diseases, with the vacA s1m1/cagA+ genotype being the most frequent [Contribution 3]. The study also revealed a 19.8% prevalence of clarithromycin resistance-associated mutations. These findings suggest the need for routine testing for *H. pylori* virulence factors and clarithromycin resistance-associated mutations to guide treatment decisions. In Japan, a study investigated the serotypes and antibiotic resistance of Streptococcus pneumoniae before and after the introduction of the 13-Valent Pneumococcal Conjugate Vaccine [Contribution 4]. The key finding is that cefotaxime resistance was confirmed for the serotype 15A isolates. Future trends in the spread of these isolates should be monitored with caution. 

In addition, a comprehensive analysis of SARS-CoV-2 outlines the genomic structure of the virus, demonstrating its close relation to bat-derived coronaviruses, suggesting a zoonotic origin [3]. Researchers explored the virus’s receptor-binding mechanisms, highlighting similarities with SARS-CoV, which indicates that the virus utilizes the same receptor, ACE2, for cell entry. The study underscores the virus’s transmission dynamics and potential cross-species transfer, laying a foundation for developing effective diagnostics, treatments, and vaccines to combat the spread of the disease, which would later be designated as COVID-19. These studies highlight the urgent need for robust epidemiological surveillance and research to understand disease transmission dynamics, inform public health interventions, and protect global health. As we navigate the post-COVID-19 world, studying the epidemiology of pathogenic agents will continue to be paramount.

## 2. Tuberculosis and Hematological Parameters

Tu et al. indicated that these parameters displayed a substantial discriminatory power in differentiating between PTB and GUTB [Contribution 5], suggesting they could serve as potential markers for these tuberculosis types. Specifically, the PTB patients exhibited higher NLR, whereas GUTB patients demonstrated increased lymphocyte and monocyte ratios, indicative of maintained cell-mediated acquired immunity. This finding aligns with others [4,5] and highlights the significance of analyzing immunological markers, hematological parameters, and biochemical profiles in tuberculosis patients for diagnostic accuracy, disease differentiation, and tailored treatment approaches. The implications include incorporating these parameters into routine assessments to enhance tuberculosis diagnosis, patient management, and treatment outcomes. Development of more targeted diagnostic strategies, personalized treatment plans based on disease manifestations, and improved monitoring techniques for patients with different forms of tuberculosis may be applied for better patient outcomes. Incorporating immunological, biochemical, and hematological parameters into clinical practice could lead to more precise and effective tuberculosis management, ultimately contributing to better patient outcomes and disease control strategies.

## 3. Outbreak Investigation and Virology

An epidemiological, clinical, and virological study was conducted on the first four cases of Monkeypox (Mpox) found in Cartagena, Colombia, during the 2022 outbreak through passive surveillance [Contribution 6]. Since Monkeypox was first isolated in 1958 from laboratory monkeys, genomic studies have characterized MPXV into Central African/Congo Basin and West Africa clades with differential epidemiology and clinical manifestations [6,7]. All cases in the study tested positive for MPXV, specifically identifying clade IIB and lineage B.1 from the genetic sequencing. The study yielded vital genomic, clinical, and epidemiological data that deepen the understanding of Mpox at a local level, emphasizing Cartagena’s susceptibility due to its significant domestic and international connectivity. Furthermore, the research suggests that multiple introduction events possibly happened in Cartagena, with transmission likely occurring through skin-to-skin sexual contact.

In addition, a retrospective study was conducted in Romania to assess the prevalence of bacterial and fungal co- and superinfections in COVID-19 patients [Contribution 7]. The study found that *Pseudomonas aeruginosa* and *Klebsiella pneumoniae* were the most common pathogens identified in sputum samples, followed by *Escherichia coli* and *Acinetobacter baumannii*. Patients with fungal co-infections were noted to have a shorter duration between symptom onset and hospitalization, elevated lymphocyte counts and transaminase levels, and more severe complications at the time of admission compared to others. Furthermore, these patients showed lower oxygen saturations and a higher rate of mortality, particularly when the infections were multidrug-resistant. The authors emphasized the urgent need for stringent antimicrobial stewardship and infection control policies in Romania, particularly considering the widespread use of antimicrobial agents and the increased antibiotic resistance during the COVID-19 pandemic, exacerbated by over-the-counter antibiotic access.

## 4. Co-Infections and Community Health

A study assessed the prevalence of single and multiple diarrheal-causing pathogen combinations in children from rural and peri-urban communities in South Africa [Contribution 8]. A total of 275 diarrhea stool specimens were collected and analyzed using the BioFire® FilmArray® Gastrointestinal panel. The results showed that 82% of the specimens contained enteric pathogens. The most detected bacterial, viral, and parasitic pathogens were EAEC (42%), EPEC (32%), Adenovirus F40/41 (19%), Norovirus (15%), Giardia (8%), and Cryptosporidium (6%), respectively. Single enteric pathogen infections were recorded in 24% of the specimens, while multiple enteric pathogen combinations were recorded in 59%. The study demonstrated the complex nature of pathogen co-infections in diarrheal episodes, which could impact treatment effectiveness. Additionally, understanding the prevalence and combinations of co-infections could inform public health interventions to prevent diarrheal diseases, particularly in vulnerable populations such as young children in rural and peri-urban communities.

## 5. COVID-19 and Dermatological Manifestations

A case study was presented to discuss a patient with genetic thrombophilia and a mutation in the MTHFR gene in Africa [Contribution 9]. Treatment with rivaroxaban and prednisone led to the resolution of dermatological symptoms and decreased D-dimer levels, indicating reduced blood clot formation. The patient was also diagnosed with COVID-19, caused by the P.2 variant of SARS-CoV-2. This case study highlights the importance of considering genetic predispositions and thrombophilic tendencies in patients with COVID-19, as these factors may influence disease presentation and outcomes, including dermatological symptoms. Future research in this area could focus on elucidating the underlying mechanisms by which genetic thrombophilic conditions interact with SARS-CoV-2 infection to manifest dermatological symptoms, as well as exploring potential implications for personalized treatment approaches and disease management strategies in thrombophilic individuals with COVID-19. Further investigations into the association between thrombophilic mutations and dermatological manifestations in COVID-19 could enhance our understanding of disease pathogenesis and inform targeted interventions for this specific patient population. However, further research is needed due to the limitations of a single case study.

In the field of epidemiology and pathogenic agents, more research and funding are much needed. For example, there is an increasing prevalence of infections caused by drug-resistant infectious agents and the development of bacteriocins, a group of antimicrobial peptides produced by bacteria, as a potential solution [8]. The urgency to increase and advance the in vivo models that both assess the efficacy of bacteriocins as antimicrobial agents and evaluate possible toxicity and side effects, which are key factors to determine their success as potential therapeutic agents in the fight against infections caused by multidrug-resistant microorganisms, has been noted. Furthermore, plant extracts, essential oils, small antimicrobial peptides of animal origin, bacteriocins, and various groups of plant compounds (triterpenoids, alkaloids, phenols, flavonoids) have shown antimicrobial and antiviral activity [9,10]. Many existing studies utilize in silico and in vitro testing as an initial approach to ascertain the health advantages of natural products, both with and without a carrier system. In vitro tests might demonstrate the potential antimicrobial effect of the complex formed by the natural product and its delivery system. However, these findings should be supplemented with toxicity tests to prevent severe side effects. Subsequently, in vivo studies should be conducted using the most appropriate animal models. These studies aim to determine if certain compounds in the body could inhibit, block, degrade, or interfere with the drug.

Last but not least, the World Health Organization (WHO) is initiating a global scientific process to update the list of priority pathogens, which are agents that can cause outbreaks or pandemics. This process aims to guide global investment, research, and development, particularly in vaccines, tests, and treatments. Over 300 scientists will consider evidence on over 25 virus families and bacteria, as well as “Disease X”, an unknown hypothetical pathogen that could cause a serious international epidemic [11]. The current list of priority pathogens includes COVID-19, Crimean–Congo hemorrhagic fever, Ebola virus disease, Marburg virus disease, Lassa fever, Middle East respiratory syndrome (MERS), Severe Acute Respiratory Syndrome (SARS), Nipah and henipaviral diseases, Rift Valley fever and Zika [12]. The WHO develops R&D roadmaps and target product profiles and facilitates clinical trials for these priority pathogens. The list has become a reference point for the research community to manage future threats. It is important to note that the field of epidemiology is dynamic and constantly evolving. New pathogens can emerge and known pathogens can develop new resistance patterns. Therefore, ongoing surveillance and research are crucial for early detection and response to potential epidemics.
